# Enhancement of polymer endurance to UV light by incorporation of semiconductor nanoparticles

**DOI:** 10.1186/s11671-015-0787-5

**Published:** 2015-02-26

**Authors:** Galyna Rudko, Andrii Kovalchuk, Volodymyr Fediv, Weimin M Chen, Irina A Buyanova

**Affiliations:** V. Lashkaryov Institute of Semiconductor Physics of National Academy of Sciences of Ukraine, 45, Pr. Nauky, Kiev, 03028 Ukraine; Department of Biophysics and Medical Informatics, Bukovinian State Medical University, 42 Kobylyanska st., 58000 Chernivtsi, Ukraine; Department of Physics, Chemistry and Biology, Linköping University, SE-581 83 Linköping, Sweden

**Keywords:** Polymer, Nanoparticles, PVA, CdS, Shielding, Nanocomposite

## Abstract

Improvement of polyvinyl alcohol stability against ultraviolet (UV) illumination is achieved by introducing cadmium sulfide (CdS) nanoparticles into the polymeric matrix. Enhancement of stability is analyzed by optical characterization methods. UV protection is achieved by diminishing the probability of photo-activated formation of defects in polymer. The sources of polymer protection are the lowering of the efficiency of polymer excitation via partial absorption of incident light by the embedded nanoparticles as well as the de-excitation of the macromolecules that have already absorbed UV quanta via energy drain to nanoparticles. Within the nanoparticles, the energy is either dissipated by conversion to the thermal energy or reemitted as visible-range photoluminescence quanta.

## Background

Polymers are widely used materials in a large variety of commercial and technical applications [1]. Natural and synthetic polymers can be produced with a diversity of physical properties. Among a variety of physical properties that ensure the uniqueness and benefits of polymers, it is necessary to highlight their flexibility, good mechanical properties, stiffness, strength, heat resistance, etc. [2]. However, the applications of polymers are essentially narrowed down by the ability of photo-degradation, especially, under the ultraviolet (UV) light [3]. Polymers deteriorate due to the effect of UV-stimulated photo-oxidative reactions. Typically, photo-ageing involves absorption of quanta of UV light by impurities which in turn release a sufficient energy to cause bond scission and formation of free radicals or chromophores. The degradation of polymers starts with visible color changing and further leads to cracking and hazing. These changes can be detected by optical methods (absorption, photoluminescence) and could serve as photo-ageing indicators.

In order to reduce the harmful effects of UV light, two types of protectors could be used. The first type is organic UV screeners (absorbers) that have an increased ability to absorb light in the UV range with further transmitting UV energy to thermal one. This reduces an amount of UV light that can be absorbed by chromophores present in the polymer. The second type is organic UV stabilizers; their molecules work as radical scavengers that are able to deactivate the products of photolysis effectively inhibiting the destructive effects of photo-oxidation, i.e., they reduce the number of photo-catalytic reaction products [4].

However, these methods of UV screening employ organic materials, which in their turn are changing under the influence of UV light. Even if their change does not spoil the source material, durability of protective action is limited, which devaluates their usefulness.

Recently, a new type of inorganic UV absorbers, based on semiconductor nanoparticles, was also used as efficient UV screeners in polymeric systems [5]. The incorporation of semiconductor nanoparticles (NPs) into polymers leads to dramatic changes in optical properties of the polymer [6]. NPs usually have higher photostability than organic molecules and are, therefore, of great interest as additives to polymers. Moreover, it is possible to change the absorption edge of the composite via tuning the particle size.

In the present study, we have focused on the UV protection of polyvinyl alcohol (PVA). This polymer plays an important role in industrial applications such as fiber and textile sizing, coating, adhesives, emulsifiers, film packaging of food, etc., because of its strong biodegradability, water solubility, good film-forming properties, and chemical resistance [7]. Unfortunately, like other organic materials, it is sensitive to UV light that is the major limitation for its use in exterior conditions. Semiconductor cadmium sulfide (CdS) NPs have been chosen as a stabilizing antiageing improver, and their influence on the UV endurance of the NP-containing polymer was studied by optical methods.

## Methods

### Nanocomposite fabrication

The nanocomposite films of CdS/PVA were fabricated by the colloidal chemical route. Colloidal solutions of CdS NPs were synthesized starting with 5% water solution of PVA as a base. CdCl_2_ and Na_2_S salts were used as precursors for the NP growth. To maintain a constant supply of the building material for growing NPs, the concentration of the precursors in the growth solution was kept at a constant level by multiple stepwise additions of the precursors to the growth solution. Concentrations of precursors and pH values were kept within the limits estimated by analyzing the probabilities of possible chemical reactions to avoid formation of Cd(OH)_2_ instead of CdS. By this procedure, NPs were synthesized directly in PVA and the macromolecules of PVA played a role of capping agents that restricted NP sizes during the growth. All chemicals were of analytical grade and were used without any further purification.

Thin solid nanocomposite films of CdS/PVA were formed by casting from aqueous solution and drying in a pressure-tight vessel containing an absorbent.

All steps of composite production were carried out under ambient conditions.

### UV treatment and characterization methods

A Coherent Verdi 2-W laser operating at *λ*_exc_ = 266 nm was used as a source of UV light. Treatments were done with an unfocused laser beam that was passed through a round diaphragm with the diameter of 5 mm that was used to restrict the size of the illuminated spot on the sample. Total power of the laser beam after the diaphragm was 50 mW. Duration of the UV treatment was 2 h.

Optical transmission measurements of untreated and UV-exposed samples were carried out using two types of light sources: an XBO lamp and a visible-light lamp were used for the measurements of absorption in the UV range of the spectrum (250 to 400 nm) and in the visible range of the spectrum (400 to 750 nm), respectively. In both cases, a double monochromator (SPEX-1404 0.85 m) combined with a PMT detector was used for spectral dispersion and detection.

For the photoluminescence (PL) measurements, the same laser as for the UV treatment was used. Usage of the UV light for excitation puts the constraint on the experimental conditions. To avoid the deterioration of sample properties during the measurements, one has to use as low power as possible. Thus, the power of the laser beam was minimized to 0.5 mW. As a result, the UV flux during PL spectra acquisition was by approximately seven times lower than during the UV treatment and, respectively, the total energy of UV exposure was by approximately 5 orders of magnitude lower.

PL spectra were recorded by a Princeton Instruments ST-133 CCD camera (Princeton Instruments, Trenton, NJ, USA) that was attached to a 0.5 m Acton SpectraPro 2500i monochromator.

Both UV treatment and all optical measurements were done at ambient conditions.

## Results and discussion

**Figure 1:**
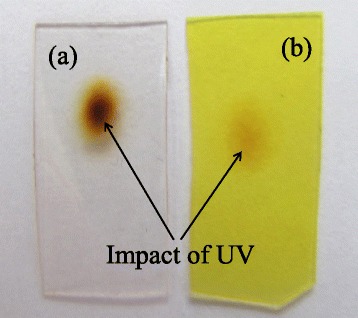
**The fotos of the samples. (a)** Unloaded polymer and **(b)** CdS/PVA nanocomposite subjected to the UV light. The spots on the samples are the result of the UV-laser impact.

Figure 1(a,b) shows photos of pure PVA (a) and CdS/PVA nanocomposite (b) films after UV exposure. It is seen that the sample of pure PVA is a transparent colorless film with a brownish dark circle spot (shown by the arrow in Figure 1(a)) that appears after the illumination with UV light. The coloring within the spot is uneven. Its variation reflects spatial distribution of the intensity within the laser beam cross-section, and the darker central part of the circle corresponds to the most intense central part of the laser beam.

The sample of CdS/PVA nanocomposite is a light-yellow film. The trace of the UV laser on it shows up as a spot of a little bit darker yellow shade (shown by the arrow in Figure 1(b)). The comparison of the two photos clearly demonstrates that the impact of UV illumination is much weaker for CdS/PVA nanocomposite. Note that the concentration of NPs in the composite is only 4 · 10^16^ сm^−3^. The latter was estimated using i) total amounts of Cd and S precursors added to the growth solution, ii) the average size of NPs (approximately 5.3 nm) calculated from the absorption edge of the untreated composite by Brus formula [8], and iii) CdS lattice constant of 0.58 nm.

**Figure 2:**
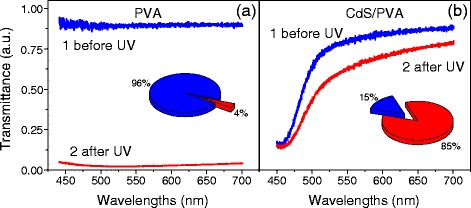
**Spectral dependences of the transmittance of the samples in the visible range.** Unloaded polymer **(a)** and CdS/PVA nanocomposite **(b)**. Curves 1 and 2 correspond to the untreated sample and the UV-exposed sample, respectively. Diagrams in the inserts show the relative changes of the integrated visible-range transmittance of the samples caused by UV treatment: 100%, integrated transmittance before UV-exposure; 4% and 85%, integrated transmittance of PVA and CdS/ PVA after UV exposure, respectively.

These visible qualitative differences were quantitatively analyzed by optical characterization techniques. Figure 2 shows visible-range transmittance spectra of the unloaded polymer (Figure 2a) and CdS/PVA nanocomposite (Figure 2b) measured before the UV treatment and after it (within the damaged part of the sample).

In full agreement with Figure 1, the untreated PVA demonstrates high transparency in the whole visible range (curve 1, Figure 2a) which results from the wide highest occupied molecular orbital (HOMO)-lowest unoccupied molecular orbital (LUMO) gap of this polymer (of about 6 eV [9,10]). After the UV treatment, the transmittance of PVA is almost completely eliminated in the whole visible range. Curve 2 in Figure 2a demonstrates that the transmittance is almost independent of the wavelength and its absolute value varies in the range of 2 to 3%. The transmission of the sample integrated over the whole visible range drops to 4% of the initial value as is shown by the diagram in the inset in Figure 2a. This huge change in the transmittance is ascribed to two main contributions. The first is the absorption caused by new defects generated within PVA by UV light. Individual absorption bands of these defects overlap and produce strong and featureless background. Another contribution comes from increased scattering of light by UV-induced imperfections in the polymer.

The influence of the UV exposure on the transmittance of CdS/PVA nanocomposite is shown in Figure 2b. Curve 1 that corresponds to the transmittance of the untreated composite demonstrates an abrupt decrease of the transmittance at short wavelengths. This is the manifestation of the fundamental absorption edge of CdS NPs, which are incorporated into the polymer. (We note that it is the fundamental absorption of NPs that causes the light-yellow color of the composite films.) Curve 2 in Figure 2b shows the transmittance of the CdS/PVA nanocomposite after the UV illumination. It is seen that the influence of UV light on the transmittance of nanocomposite is not that strong as in the case of the unloaded polymer. The overall decrease of transmittance is predominantly caused by the appearance of spectrally independent background that becomes as high as 5 to 6% and can be ascribed, similarly to the case of pure PVA, to the increased scattering and absorption by UV-induced defects in polymer. Thus, the influence of the UV light on the composite is much lower than that on the unloaded polymer. This is also clearly seen from the diagram in the inset in Figure 2b: after the UV treatment, the integrated transmission of the nanocomposite remains at the level of 85% of the initial transmittance as compared to 4% in the case of the unloaded polymer.

**Figure 3:**
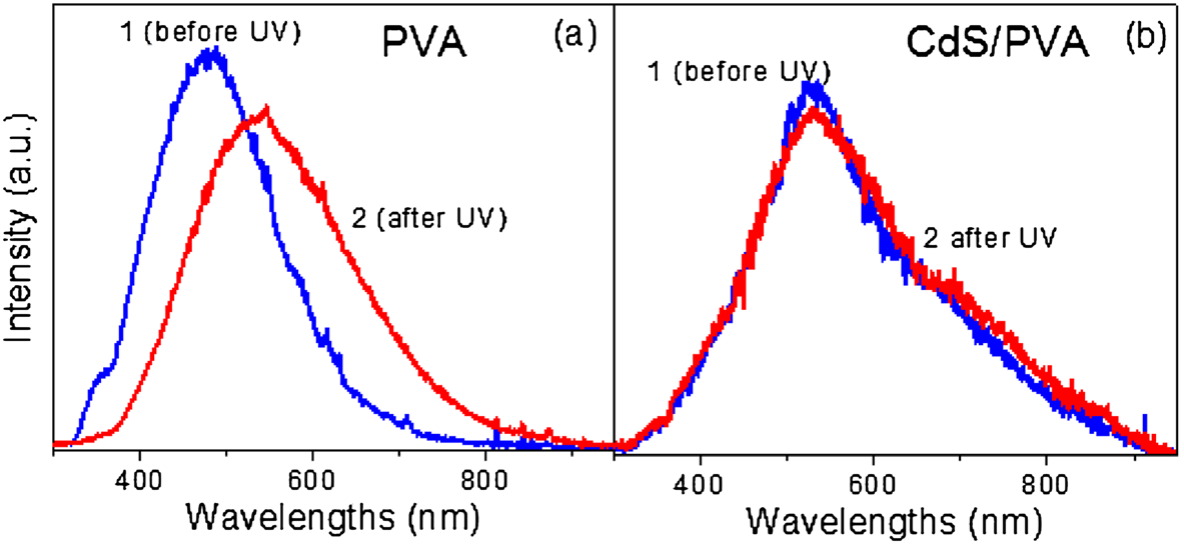
**PL spectra. (a)** Unloaded polymer and **(b)** CdS/PVA nanocomposite. Curves 1 and 2 correspond to the untreated sample and the UV-exposed sample, respectively.

Information about the UV-induced changes in the samples under the study can also be obtained from PL measurements. Figure 3 shows the variation of light-emitting properties of the polymer (a) and nanocomposite (b) under the UV exposure. The UV treatment of the unloaded PVA causes a very strong red shift (about 70 nm) of the PL band. It should be noted that the PL spectrum of the pure PVA is related to the defects of macromolecules [11,12]. Thus, the changes of the PL band reflect the UV-induced changes in the defect subsystem of the polymer [13], in full agreement with the results presented in Figure 2a.

Figure 3b shows that the PL band of the nanocomposite is almost unchanged by the UV illumination. Taking into account that the emission of CdS/PVA nanocomposite is related to the NPs [14,15], from the comparison of Figure 3a,b, one can conclude that UV light causes drastic alterations in the defect subsystem of pure composite but does not change NPs.

**Figure 4:**
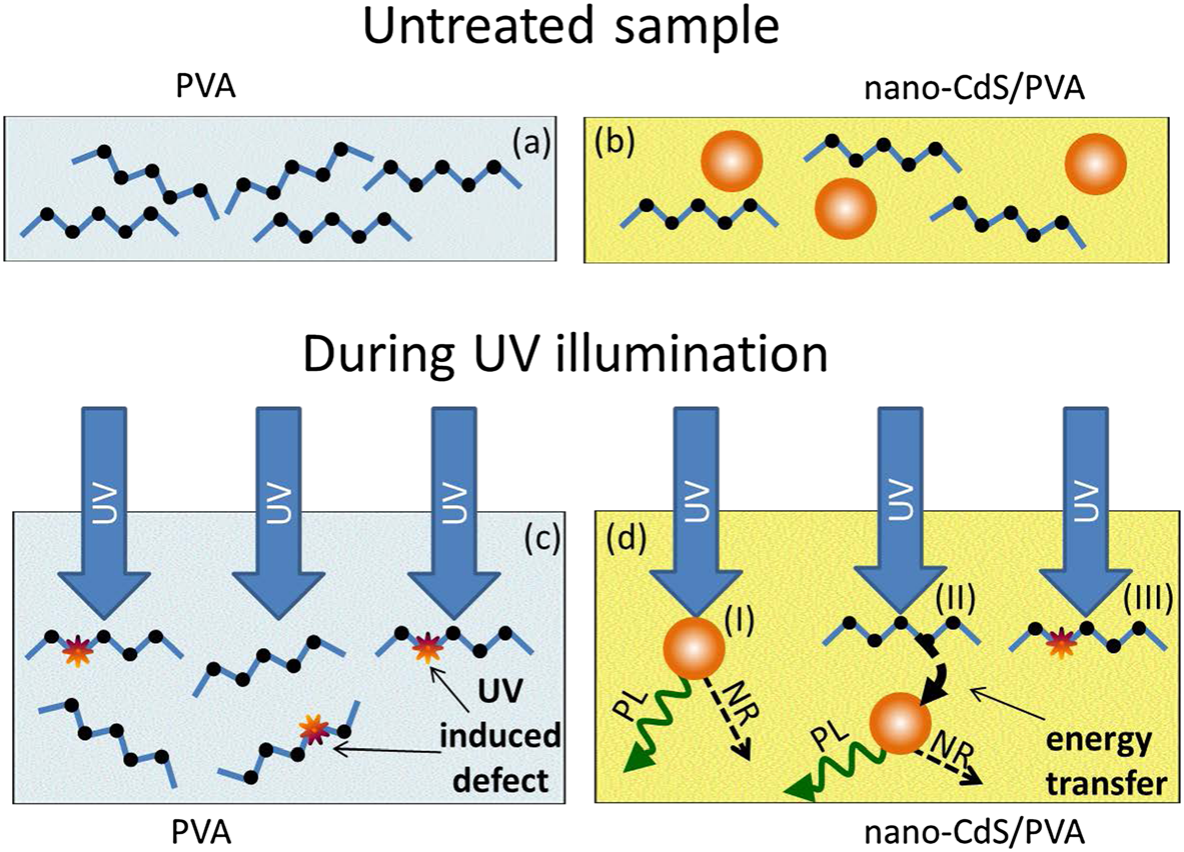
**The scheme of the UV-induced processes in the unloaded polymer (a, c) and CdS/PVA nanocomposite (b, d). (a)** Untreated unloaded PVA. The zigzag lines serve to show the macromolecules in the polymer. **(b)** Untreated nanocomposite CdS/PVA. The circles denote CdS NPs in the polymeric matrix. **(c)** Formation of the UV-induced defects in the polymer. **(d)** Processes in CdS/PVA under UV illumination: I, direct absorption of UV light by NPs and further emission of PL quanta (the process is labeled PL) or thermal dissipation of energy via nonradiative process (the process is labeled NR); II, excitation transfer from photo-excited polymer macromolecules to NPs with further emission of PL quanta (the process is labeled PL) or thermal dissipation of energy (the process is labeled NR); III, the same process as in unloaded PVA: formation of the UV-induced defects in the matrix of nanocomposite.

The experimental facts illustrated by Figures 1, 2, and 3 show that by incorporating NPs into the polymeric matrix, one can minimize the harmful influence of UV exposure on the polymeric component of the composite. In what follows, we will discuss possible mechanisms of NP participation in polymer protection and illustrate them by the schemes in Figure 4.

The energy of the exciting UV quanta used for the treatment of the samples is less than the HOMO-LUMO gap energy of PVA, and thus, the light used for the UV treatment corresponds to the transparency window of the ideal polymer. Nevertheless, the UV light with the 266-nm wavelength is absorbed by PVA due to the presence of imperfections of the polymeric chain. These defects are predominantly acetate residuals, due to incomplete hydrolysis of polyvinyl acetate used for PVA fabrication. It is known that the excitation of macromolecules typically initiates various types of photo-activated reactions in polymers, such as chain scission [16,17], formation of chromophore groups and free radicals [18-21], breaking of intermolecular bonds [17], configurational transformations [22], etc. In full agreement with these earlier observations, our transmittance and PL results demonstrate (Figures 2 and 3) the appearance in the unloaded PVA of new UV-induced moieties that absorb, scatter, and emit light (see Figure 4c). According to [13], the dominant UV-induced processes in PVA are the formation of the chromophore groups and free radicals, for example, carbonyl groups (C = O), carboxyl groups (−COOH), polyenes of various lengths, etc.

Similar photo-activated reactions can be expected in the polymeric component of CdS/PVA nanocomposites (see process III in Figure 4d). However, the presence of NPs interferes with the influence of UV light on the matrix. The most important aspect of NP influence is a mere shielding of the polymer, i.e., partial absorption of the incident light by NPs (see process I in Figure 4d). It should be noted that the absorption coefficient of CdS at 266-nm wavelength is by 5 orders of magnitude higher than the one of the unloaded PVA at the same wavelength. That is why, in spite of nanometer sizes and a low concentration of NPs, a considerable share of the UV flux is absorbed by them and not by the polymeric matrix. This diminishes the efficiency of composite matrix excitation as compared to the excitation of the same polymer with no NPs. As the result, the degradation rate must be lower for the composite as compared to the virgin PVA.

The energy of the UV radiation that is absorbed directly by NPs is either thermally dissipated or released via radiation of photoluminescence quanta. The corresponding processes are labeled in Figure 4d as NR and PL, respectively. In this way, the NPs serve as a bypass for the UV energy.

Beside the shielding of the matrix, the presence of NPs also provides de-excitation of the polymeric macromolecules that additionally decreases the probability of photo-activated reactions. The de-excitation of the macromolecules occurs in the following way. When a macromolecule is excited by UV light, the excitation easily migrates along the polymeric chain until it encounters moieties with a lower energy. Because of the much narrower band gap of CdS as compared to the HOMO-LUMO gap of PVA, NPs can serve as an energy drain for excitation (see process II in Figure 4d). The occurrence of energy transfer from the polymeric matrix to CdS NPs has been demonstrated by us earlier [23] from time-resolved PL measurements of CdS/PVA composites. It was shown that the PL emission of NPs can be excited via both the direct absorption of UV light by NPs and excitation transfer from the matrix.

## Conclusions

We have studied the influence of CdS NPs incorporation into PVA on the stability of this polymer against UV irradiation. It is shown that the presence of NPs strongly diminishes the harmful effects of UV light. This conclusion is based on better preserved transmission of the composite samples under UV exposure and a lack of changes in the PL spectra demonstrating that NPs remained intact. Enhancement of UV endurance results from the lowered probability of the formation of UV-induced defects in the polymeric matrix of the nanocomposite. The suggested sources of the UV-protective action of the NPs include the following: (i) shielding of the polymeric matrix from the incident radiation by NPs via absorption of UV light, i.e., the lowering of the efficiency of UV excitation of the polymer; and (ii) de-excitation of the already excited macromolecules via energy drain to NPs. Within the nanoparticles, the energy is either dissipated by conversion to the thermal energy or reemitted as visible-range photons.

## Abbreviations

PVA: polyvinyl alcohol
CdS: cadmium sulfide
PMT: photo multiplier
CCD: charged-coupled device
UV: ultraviolet
PL: photoluminescence
NP: nanoparticle
HOMO: highest occupied molecular orbital
LUMO: lowest unoccupied molecular orbital
